# Relationship between body mass index and renal function deterioration among the Taiwanese chronic kidney disease population

**DOI:** 10.1038/s41598-018-24757-6

**Published:** 2018-05-02

**Authors:** Tian-Jong Chang, Cai-Mei Zheng, Mei-Yi Wu, Tzu-Ting Chen, Yun-Chun Wu, Yi-Lien Wu, Hsin-Ting Lin, Jing-Quan Zheng, Nain-Feng Chu, Yu-Me Lin, Sui-Lung Su, Kuo-Cheng Lu, Jin-Shuen Chen, Fung-Chang Sung, Chien-Te Lee, Yu Yang, Shang-Jyh Hwang, Ming-Cheng Wang, Yung-Ho Hsu, Hung-Yi Chiou, Senyeong Kao, Yuh-Feng Lin

**Affiliations:** 1 Graduate Institute of Life Sciences, National Defense Medical Center, Taipei, Taiwan; 2 Performance Appraisal Section, Secretary Office, Shuang Ho Hospital, Taipei Medical University, Taipei, Taiwan; 3 Division of Nephrology, Department of Internal Medicine, School of Medicine, College of Medicine, Taipei Medical University, Taipei, Taiwan; 4 Division of Nephrology, Department of Internal Medicine, Shuang Ho Hospital, Taipei Medical University, Taipei, Taiwan; 5 Graduate Institute of Clinical Medicine, College of Medicine, Taipei Medical University, Taipei, Taiwan; 6 Institute of Epidemiology and Preventive Medicine, College of Public Health, National Taiwan University, Taipei, Taiwan; 7 School of Nursing, College of Nursing, Taipei Medical University, Taipei, Taiwan; 8 Kidney Disease Prevention Foundation, Taipei, Taiwan; 9 Department of Ophthalmology, Tri-Service General Hospital, National Defense Medical Center, Taipei, Taiwan; 10 Department of Critical Care Medicine, Shuang Ho Hospital, Taipei Medical University, Taipei, Taiwan; 11 School of Public Health, National Defense Medical Center, Taipei, Taiwan; 12 Department of Medical Education and Research, Kaohsiung Veterans General Hospital, Kaohsiung, Taiwan; 13 School of Public Health, College of Public Health and Nutrition, Taipei Medical University, Taipei, Taiwan; 14 Division of Nephrology, Department of Medicine, Fu-Jen Catholic University Hospital, School of Medicine, Fu-Jen Catholic University, Taipei, Taiwan; 15 Division of Nephrology, Department of Medicine, Tri-Service General Hospital, National Defense Medical Center, Taipei, Taiwan; 16 School of Public Health, Graduate Institute of Clinical Medical Science, China Medical University, Taichung, Taiwan; 17 Division of Nephrology, Kaohsiung Chang Gung Memorial Hospital, Chang Gung Medical University, Kaohsiung, Taiwan; 18 The Division of Nephrology, Changhua Christian Hospital, Changhua, Taiwan; 19 Division of Nephrology, Department of Medicine, Kaohsiung Medical University Hospital, Kaohsiung, Taiwan; 20 Division of Nephrology, Department of Internal Medicine, Cheng Kung University Medical Center, Tainan, Taiwan

**Keywords:** Health services, End-stage renal disease

## Abstract

This study investigated the characteristics of patients with different chronic kidney disease (CKD) stages according to various body mass index (BMI) categories and determined the influence of BMI in renal function deterioration. We conducted a multicenter, longitudinal cohort study based on the Epidemiology and Risk Factors Surveillance of CKD project (2008–2013) and National Health Insurance Research Database (2001–2013). A total of 7357 patients with CKD aged 20–85 years from 14 hospitals were included in the study. A higher male sex, diabetes mellitus (DM) and hypertension were noted among overweight and obese CKD patients, while more cancer prevalence was noted among underweight CKD patients. Charlson comorbidity index was significantly higher and correlated with BMI among late CKD patients. Patients with BMI < 18.5 kg/m^2^ exhibited non-significantly higher events of eGFR decline events in both early and late CKD stages than other BMI groups. BMI alone is not a determinant of CKD progression among our Taiwanese CKD patients. Obesity should be re-defined and body weight manipulation should be individualized in CKD patients.

## Introduction

Obesity, a global pandemic problem, is associated with various metabolic disorders and results in a shortened life span related to adverse health consequences. In Taiwan, the prevalence of overweight and obesity among adults was reported to be 44.1%, of whom 50.8% were men and 36.9% were women, according to 2005–2008 data^1^. Moreover, in a survey performed in 2002 and 2007 (Taiwanese Survey on Hypertension, Hyperglycemia, and Hyperlipidemia), the prevalence of obesity increased from 19.2% to 23.3% among men and from 13.4% to 19.0% among women^2^. The overweight and obesity rates in Taiwan are substantially higher than those in Japan, Singapore, and other neighboring Asian countries. On the other hand, the prevalence of chronic kidney disease (CKD) in Taiwan is also increasing, with nearly 6 million people undergoing dialysis and approximately 2,000 people newly diagnosed as having end-stage renal disease (ESRD) annually (http://www.tsn.org.tw/UI/K/K008.aspx). Since the numbers of obese patients with CKD and those undergoing dialysis are also increasing in parallel, the diagnosis and precise management of obesity have become critical among these patients.

Many studies have demonstrated that obesity is an important risk factor for incident CKD^3–7^ and increased risk of ESRD^8–12^. Paradoxically, obesity itself in CKD and ESRD has been found to be associated with more favorable outcomes^13,14^. A reverse obesity–mortality association has been consistently observed in patients with ESRD^15–17^; however, conflicting results have been observed among patients with CKD^13,18–20^. Body mass index (BMI) is a globally accepted anthropometric measure for obesity classification. Recently, many studies have questioned the accuracy of BMI in obesity and excess body fat assessment^21–23^. Whether BMI can influence the CKD progression among all stages of CKD in the Taiwanese population remains unclear.

We conducted a multicenter, longitudinal cohort study to investigate the characteristics of patients at all CKD stages (CKD stages 1–5 nondialysis [ND]) according to various BMI categories and to determine the influence of BMI in renal function deterioration by using the data from the Epidemiology and Risk Factors Surveillance of CKD project (2008–2013) and National Health Insurance Research Database (NHIRD) (2001–2013).

## Results

### Demographic characteristics of the patients

**Figure 1:**
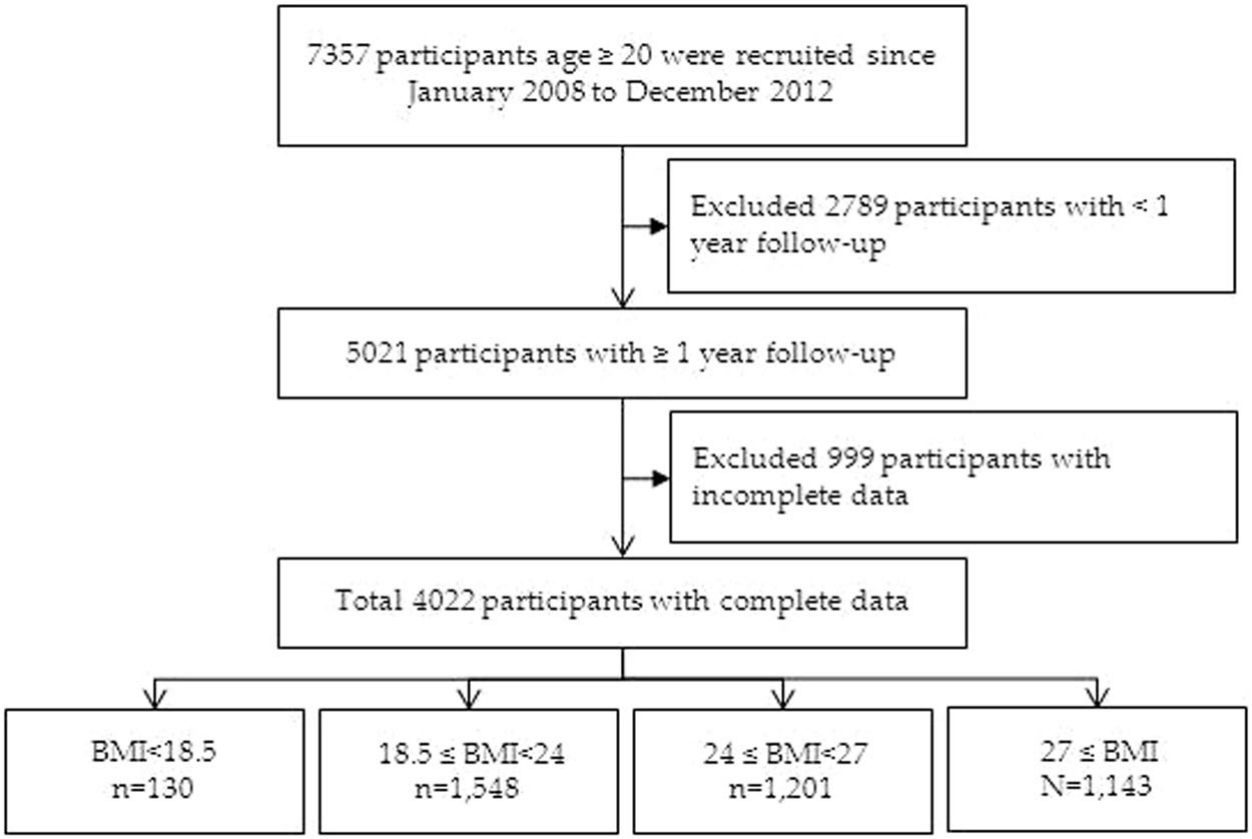
Flowchart of Patients Selection.

A total of 7357 patients with CKD aged 20–85 years from 14 hospitals were included in the study. Patients with ESRD, defined as either receiving maintenance dialysis during this period or having a kidney transplant, were excluded. After the exclusion of patients with less than 1 year of follow-up (n = 2789) and those with missing or incomplete data (n = 999), 4022 patients with CKD were finally enrolled in this study (Fig. 1). Among these patients, 2008 had early-stage CKD (CKD stages 1, 2, and 3a) and 2014 had late-stage CKD (CKD stages 3b, 4, and 5ND). The patients were continually traced from the baseline date to the end of the study period (June 18, 2015).

The mean age of the cohort was 62.86 ± 14.93 years, and 56.9% of the patients were men. The mean baseline eGFR was 51.5 ± 36.15 mL/min per 1.73 m^2^. The baseline characteristics of patients according to baseline BMI are presented in Table 1. For further analysis, we also present the baseline characteristics of patients with early-stage CKD (CKD stages 1–3a) in Table 2 and late-stage CKD (CKD stages 3b–5) in Table 3. The highest prevalence rates of overweight (24 ≤ BMI <27 kg/m^2^) and obesity (BMI ≥ 27 kg/m^2^) were observed among patients aged 45–64 years, with the values being 45% and 39.38% respectively. We also noted a male predominance in BMI (62.7% for overweight and 56.43% for obesity). Patients with higher BMI exhibited significantly higher baseline DM and hypertension than those with lower BMI (16.15% in the underweight group, compared with 36.05%, 47.29%, and 52.23% in the normal, overweight, and obesity groups, respectively). Furthermore, patients with BMI < 18.5 kg/m^2^ exhibited a characteristically higher cancer prevalence rate (18.46%) than the other groups; the prevalence rates were 9.75%, 9.16%, and 8.22% in the normal, overweight, and obesity groups, respectively. Baseline coronary artery disease (CAD) and stroke did not differ between the groups. Similarly, the baseline Charlson comorbidity index was significantly lower in the low and normal BMI groups (3.38 ± 2.50 and 3.53 ± 2.39, respectively), compared with the overweight and obesity groups (3.81 ± 2.46 and 3.73 ± 2.41, respectively). Furthermore, the higher BMI groups had higher medication use and more smoking, alcohol consumption, and betel nut chewing habits. The biochemical analysis revealed significantly higher hemoglobin and hematocrit levels, AC sugar, triglyceride, and uric acid levels in the higher BMI groups. No significant difference was observed in baseline cholesterol, electrolytes, albumin, UPCR, and eGFR between the groups (Table 1). We subgrouped our cohort into early- and late-stage CKD (CKD stages 1–3a and 3b–5) and studied their characteristics according to different BMI categories (Tables 2 and 3). Similar age and sex prevalence rates were observed after subgrouping. Among patients with early-stage CKD, those in the lower BMI group exhibited significantly higher cancer prevalence (16.67% in with the underweight group) than those in the higher BMI groups (8.64% in the normal, 8.95% in the overweight, and 6.76% in the obesity groups; p = 0.032). However, among patients with late-stage CKD, those in the lower BMI group had nonsignificantly higher cancer prevalence than those in the higher BMI groups (20% in the underweight group vs. 10.8% in the normal group, 11.39% in the overweight group, and 9.8% in the obesity group; p = 0.08). Higher DM and hypertension rates were observed in overweight and obese patients with early- and late-stage CKD. The Charlson comorbidity index did not differ significantly with BMI among patients with early-stage CKD (2.57 ± 2.31 in the underweight group vs. 2.69 ± 2.12 in the normal group, 2.92 ± 2.19 in the overweight group, and 2.86 ± 2.12 in the obesity group; p = 0.1773). However, a significantly higher Charlson comorbidity index was observed among overweight and obese patients with late-stage CKD (4.07 ± 2.47 in the underweight group vs. 4.32 ± 2.37 in the normal group, 4.71 ± 2.39 in the overweight group, and 4.67 ± 2.35 in the obesity group; p = 0.003). The initial stages of CKD did not differ significantly among patients with early-stage CKD; by contrast, they differed significantly among patients with late-stage CKD according to BMI (p = 0.0004). More prevalent CKD stage 3b (18.57% in the underweight group vs. 26.51% in the normal group, 33.3% in the overweight group, and 31.58% in the obesity group) and stage 4 (28.5% in the underweight group vs. 36.43% in the normal group, 36.35% in the overweight group, and 38.11% in the obesity group) were observed among patients in the higher BMI group; by contrast, 52.8% of patients in the underweight group had CKD stage 5 compared with 37.06%, 30.32%, 30.31% in the normal, overweight, and obesity groups, respectively.

**Table 1:** Baseline characteristics of study cohort according to different BMI.

**Characteristic**	**BMI < 18.5**	**18.5 ≤ BMI <24**	**24 ≤ BMI <27**	**27 ≤ BMI**	P value*
	**n = 130**	**n = 1548**	**n = 1201**	**n = 1143**	
Age, mean (SD), y	59.69 ± 18.55	62.92 ± 14.62	64.57 ± 13.24	60.83 ± 13.29	<0.0001
20–44	29 (22.31)	179 (11.56)	92 (7.66)	145 (12.69)	<0.0001
45–64	39 (30.00)	617 (39.86)	473 (39.38)	517 (45.23)	
65–74	27 (20.77)	388 (25.06)	343 (28.56)	323 (28.26)	
≥75	35 (26.92)	364 (23.51)	293 (24.40)	158 (13.82)	
Male sex	50 (38.46)	843 (54.46)	753 (62.70)	645 (56.43)	<0.0001
Comorbid conditions before the date index					
DM	21 (16.15)	558 (36.05)	568 (47.29)	597 (52.23)	<0.0001
CAD	3 (2.31)	37 (2.39)	42 (3.50)	35 (3.06)	0.3608
Stroke	14 (10.77)	264 (17.05)	226 (18.82)	194 (16.97)	0.1147
Cancer	24 (18.46)	151 (9.75)	110 (9.16)	94 (8.22)	0.0021
Hypertension	58 (44.62)	1109 (71.64)	982 (81.77)	985 (86.18)	<0.0001
TB	6 (4.62)	29 (1.87)	18 (1.50)	15 (1.31)	0.0411
COPD	32 (24.62)	321 (20.74)	288 (23.98)	271 (23.71)	0.1410
Charlson comorbidity index					0.0006
≤3	75 (57.69)	894 (57.75)	601 (50.04)	600 (52.49)	
4–5	37 (28.46)	368 (23.77)	341 (28.39)	285 (24.93)	
>5	18 (13.85)	286 (18.48)	259 (21.57)	258 (22.57)	
Mean (SD)	3.38 ± 2.50	3.53 ± 2.39	3.81 ± 2.46	3.73 ± 2.41	0.0086
ACEI used within 1 year before the index date	18 (13.85)	245 (15.83)	204 (16.99)	195 (17.06)	0.6507
ARB used within 1 year before the index date	49 (37.69)	778 (50.26)	689 (57.37)	736 (64.39)	<0.0001
fibrate used within 1 year before the index date	0 (0.00)	94(5.60)	115 (9.58)	138(12.07)	<0.0001
OHAs used within 1 year before the index date	17 (13.08)	443 (28.62)	484 (40.30)	543 (47.51)	<0.0001
Statin drug used within 1 year before the index date	20 (15.38)	430 (27.78)	380 (31.64)	423 (37.01)	<0.0001
NSAIDs used within 1 year before the index date	41 (31.54)	582 (37.60)	583 (48.54)	570 (49.87)	<0.0001
Initial stage					0.3179
1–3A	60 (46.15)	752 (48.58)	604 (50.29)	592 (51.79)	
3B–5	70 (53.85)	796 (51.42)	597 (49.71)	551 (48.21)	
Smoking	19 (14.62)	341 (22.03)	335 (27.89)	328 (28.70)	<0.0001
Alcohol consumption	8 (6.15)	140 (9.04)	119 (9.91)	150 (13.12)	0.0018
Betel nut chewing	4 (3.08)	69 (4.46)	67 (5.58)	91 (7.96)	0.0007
AC sugar	103.88 ± 34.33	110.28 ± 37.56	118.17 ± 48.87	122.35 ± 48.15	<0.0001
Cholesterol	180.73 ± 41.87	182.68 ± 43.48	184.04 ± 46.67	186.92 ± 47.79	0.1281
TG	96.35 ± 57.58	119.96 ± 71.46	146.93 ± 96.24	168.91 ± 125.71	<0.0001
Na	137.01 ± 14.01	139.62 ± 5.16	139.26 ± 6.09	141.22 ± 46.39	0.3487
K	4.82 ± 3.88	4.44 ± 0.62	4.54 ± 4.48	4.67 ± 5.31	0.5480
Ca	8.86 ± 0.74	8.98 ± 2.63	8.97 ± 0.74	9.03 ± 0.68	0.7959
P	4.17 ± 0.99	4.02 ± 1.07	3.93 ± 0.96	4.04 ± 1.53	0.2067
UA	6.71 ± 2.17	6.74 ± 2.64	7.04 ± 1.88	7.25 ± 2.69	<0.0001
Hb	11.22 ± 3.29	11.75 ± 2.53	12.31 ± 2.41	12.55 ± 2.36	<0.0001
Hct	32.69 ± 7.35	34.77 ± 6.57	36.45 ± 6.21	37.75 ± 16.11	<0.0001
Albumin	4.03 ± 0.43	4.52 ± 14.58	4.09 ± 0.50	4.15 ± 1.63	0.7929
UPCR	1180.51 ± 6016.47	3346.00 ± 90781.26	1119.61 ± 3347.45	1179.61 ± 2543.57	0.6952
eGFR	48.29 ± 38.58	50.89 ± 37.03	51.14 ± 33.75	53.07 ± 35.24	0.2775

^*^Categorical variables: chi-squared test; continuous variables: ANOVA.

DM, diabetes mellitus; CAD, coronary artery disease; CKD; chronic kidney disease; OHA, oral hypoglycemic agents; TG, total glycerides; UA, urine albumin; Hb, hemoglobin; Hct, hematocrit; UPCR, urine protein-to-creatinine ratio; eGFR, estimated glomerular filtration rate.

**Table 2:** Baseline characteristics of CKD stages 1–3a according to different BMI.

Characteristic	BMI < 18.5	18.5 ≤ BMI <24	24 ≤ BMI <27	27 ≤ BMI	P value*
	n = 60	n = 752	n = 604	n = 592	
Age, mean (SD), y	56.63 ± 18.63	59.85 ± 15.19	61.87 ± 13.59	58.25 ± 13.78	<0.0001
20–44	16 (26.67)	124 (16.49)	62 (10.26)	99 (16.72)	<0.0001
45–64	19 (31.67)	323 (42.95)	281 (46.52)	285 (48.14)	
65–74	13 (21.67)	170 (22.61)	152 (25.17)	146 (24.66)	
≥75	12 (20.00)	135 (17.95)	109 (18.05)	62 (10.47)	
Male sex	23 (38.33)	386 (51.33)	359 (59.44)	338 (57.09)	0.0008
Comorbid conditions before the date index					
DM	10 (16.67)	222 (29.52)	244 (40.40)	263 (44.43)	<0.0001
Stroke	7 (11.67)	103 (13.70)	89 (14.74)	75 (12.67)	0.7318
Cancer	10 (16.67)	65 (8.64)	42 (6.95)	40 (6.76)	0.0324
Hypertension	17 (28.33)	459 (61.04)	438 (72.52)	464 (78.38)	<0.0001
TB	3 (5.00)	10 (1.33)	7 (1.16)	9 (1.52)	0.1234
COPD	14 (23.33)	138 (18.35)	128 (21.19)	122 (20.61)	0.5001
Charlson comorbidity index					0.3261
≤3	41 (68.33)	542 (72.07)	403 (66.72)	402 (67.91)	
4–5	15 (25.00)	138 (18.35)	136 (22.52)	123 (20.78)	
>5	4 (6.67)	72 (9.57)	65 (10.76)	67 (11.32)	
Mean (SD)	2.57 ± 2.31	2.69 ± 2.12	2.92 ± 2.19	2.86 ± 2.12	0.1773
Initial stage					0.3175
1	21 (35.00)	246 (32.71)	170 (28.15)	184 (31.08)	
2	22 (36.67)	325 (43.22)	278 (46.03)	278 (46.96)	
3a	17 (28.33)	181 (24.07)	156 (25.83)	130 (21.96)	
ACEI used within 1 year before the index date	4 (6.67)	104 (13.83)	100 (16.56)	99 (16.72)	0.0950
ARB used within 1 year before the index date	16 (26.67)	310 (41.22)	292 (48.34)	331 (55.91)	<0.0001
fibrate used within 1 year before the index date	0 (0.00)	35 (4.65)	53 (8.77)	63 (10.64)	<0.0001
OHAs used within 1 year before the index date	8 (13.33)	172 (22.87)	207 (34.27)	233 (39.36)	<0.0001
Statin drug used within 1 year before the index date	9 (15.00)	193 (25.66)	172 (28.48)	199 (33.61)	0.0012
NSAIDs used within 1 year before the index date	21 (35.00)	264 (35.11)	285 (47.19)	295 (49.83)	<0.0001
Smoking	9 (15.00)	144 (19.15)	150 (24.83)	157 (26.52)	0.0032
Alcohol consumption	7 (11.67)	83 (11.04)	65 (10.76)	90 (15.20)	0.0683
Betel nut chewing	4 (6.67)	26 (3.46)	31 (5.13)	29 (4.90)	0.3408
Fasting sugar	101.10 ± 28.48	109.88 ± 41.03	118.80 ± 49.11	121.97 ± 50.23	<0.0001
Cholesterol	192.11 ± 35.56	187.66 ± 45.76	187.38 ± 47.18	185.50 ± 46.85	0.7213
TG	94.37 ± 69.46	112.97 ± 65.85	142.75 ± 98.22	162.65 ± 137.09	<0.0001
Na	139.11 ± 3.36	139.70 ± 3.42	139.35 ± 9.21	139.58 ± 9.34	0.9546
K	4.17 ± 0.55	4.20 ± 0.46	4.58 ± 7.36	4.63 ± 7.91	0.7857
Ca	9.41 ± 0.85	9.33 ± 5.05	9.09 ± 0.66	9.20 ± 0.67	0.8441
P	3.54 ± 0.65	3.58 ± 0.80	3.43 ± 0.58	3.47 ± 0.59	0.1372
UA	5.89 ± 2.01	5.89 ± 1.54	6.33 ± 1.55	6.67 ± 3.29	<0.0001
Hb	13.23 ± 4.02	13.15 ± 2.42	13.41 ± 1.67	13.75 ± 1.69	<0.0001
Hct	37.38 ± 6.33	38.80 ± 5.43	39.93 ± 4.53	41.86 ± 21.99	0.0008
Albumin	4.04 ± 0.53	4.04 ± 1.17	4.18 ± 0.55	4.40 ± 2.56	0.0883
UPCR	1330.92 ± 8727.33	660.08 ± 2620.22	443.87 ± 1304.68	646.90 ± 2018.90	0.0520
eGFR	82.94 ± 29.17	81.67 ± 28.96	78.57 ± 24.77	81.13 ± 25.26	0.1453

^*^Categorical variables: chi-squared test; continuous variables: ANOVA.

BMI, body mass index; DM, diabetes mellitus; CAD, coronary artery disease; CKD; chronic kidney disease; OHA, oral hypoglycemic agents; TG, total glycerides; UA, urine albumin; Hb, hemoglobin; Hct, hematocrit; UPCR, urine protein-to-creatinine ratio; eGFR, estimated glomerular filtration rate.

**Table 3:** Baseline characteristics of CKD stages 3b-5 according to different BMI.

Characteristic	BMI < 18.5	18.5 ≤ BMI < 24	24 ≤ BMI <27	27 ≤ BMI	*P* value*
	*n* = 70	*n* = 796	*n* = 597	*n* = 551	
Age, mean (SD), y	62.31 ± 18.21	65.81 ± 13.44	67.3 ± 12.29	63.59 ± 12.17	<0.0001
20–44	13 (18.57)	55 (6.91)	30 (5.03)	46 (8.35)	<0.0001
45–64	20 (28.57)	294 (36.93)	192 (32.16)	232 (42.11)	
65–74	14 (20.00)	218 (27.39)	191 (31.99)	177 (32.12)	
≥75	23 (32.86)	229 (28.77)	184 (30.82)	96 (17.42)	
Male sex	27 (38.57)	457 (57.41)	394 (66.00)	307 (55.72)	<0.0001
Comorbid conditions before the date index					
DM	11 (15.71)	336 (42.21)	324 (54.27)	334 (60.62)	<0.0001
Stroke	7 (10.00)	161 (20.23)	137 (22.95)	119 (21.60)	0.0765
Cancer	14 (20.00)	86 (10.80)	68 (11.39)	54 (9.80)	0.0817
Hypertension	41 (58.57)	650 (81.66)	544 (91.12)	521 (94.56)	<0.0001
TB	3 (4.29)	19 (2.39)	11 (1.84)	6 (1.09)	0.1720
COPD	18 (25.71)	183 (22.99)	160 (26.80)	149 (27.04)	0.2785
Charlson comorbidity index					0.0001
≤3	34 (48.57)	352 (44.22)	198 (33.17)	198 (35.93)	
4–5	22 (31.43)	230 (28.89)	205 (34.34)	162 (29.4)	
>5	14 (20.00)	214 (26.88)	194 (32.50)	191 (34.66)	
Mean (SD)	4.07 ± 2.47	4.32 ± 2.37	4.71 ± 2.39	4.67 ± 2.35	0.0032
Initial stage					0.0004
3b	13 (18.57)	211 (26.51)	199 (33.33)	1748 (31.58)	
4	20 (28.57)	290 (36.43)	217 (36.35)	210 (38.11)	
5	37 (52.86)	295 (37.06)	181 (30.32)	167 (30.31)	
ACEI used within 1 year before the index date	14 (20.00)	141 (17.71)	104 (17.42)	96 (17.42)	0.9583
ARB used within 1 year before the index date	33 (47.14)	468 (58.79)	397 (66.50)	405 (73.50)	<0.0001
fibrate used within 1 year before the index date	0 (0.00)	59 (6.81)	62 (10.39)	75 (13.61)	0.0001
OHAs used within 1 year before the index date	9 (12.86)	271 (34.05)	277 (46.40)	310 (56.26)	<0.0001
Statin drug used within 1 year before the index date	11 (15.71)	237 (29.77)	208 (34.84)	224 (40.65)	<0.0001
NSAIDs used within 1 year before the index date	20 (28.57)	318 (39.95)	298 (49.92)	275 (49.91)	<0.0001
Smoking	10 (14.29)	197 (24.75)	185 (30.99)	171 (31.03)	0.0012
Alcohol consumption	1 (1.43)	57 (7.16)	54 (9.05)	60 (10.89)	0.0144
Betel nut chewing	0 (0)	43 (5.40)	36 (6.03)	62 (11.25)	<0.0001
Fasting sugar	106.29 ± 38.81	110.62 ± 34.29	117.57 ± 48.68	122.75 ± 45.95	<0.0001
Cholesterol	170.53 ± 44.69	178.05 ± 40.73	180.6 ± 45.95	188.42 ± 48.77	0.0002
TG	97.98 ± 46.07	126.49 ± 75.81	151.19 ± 94.09	175.51 ± 112.21	<0.0001
Na	136.31 ± 16.04	139.58 ± 5.75	139.21 ± 3.36	142.17 ± 57.76	0.3459
K	5.03 ± 4.46	4.56 ± 0.66	4.52 ± 0.64	4.70 ± 2.73	0.0643
Ca	8.7 ± 0.62	8.86 ± 0.72	8.92 ± 0.76	8.96 ± 0.67	0.0123
P	4.33 ± 1.01	4.16 ± 1.11	4.13 ± 1.00	4.27 ± 1.72	0.2748
UA	7.34 ± 2.1	7.51 ± 3.16	7.74 ± 1.93	7.85 ± 1.68	0.0597
Hb	9.9 ± 1.73	10.64 ± 2.02	11.36 ± 2.55	11.43 ± 2.34	<0.0001
Hct	29.62 ± 6.3	31.77 ± 5.69	33.64 ± 5.97	34.18 ± 6.14	<0.0001
Albumin	4.03 ± 0.39	4.73 ± 17.58	4.04 ± 0.47	4.00 ± 0.49	0.6969
UPCR	1051.59 ± 1584.35	5883.46 ± 126558.14	1803.27 ± 4461.86	1751.97 ± 2901.74	0.7267
eGFR	18.59 ± 10.75	21.81 ± 11.53	23.38 ± 11.79	22.92 ± 11.62	0.0019

^*^Categorical variables: chi-squared test; continuous variables: ANOVA.

BMI, body mass index; DM, diabetes mellitus; CAD, coronary artery disease; CKD; chronic kidney disease; OHA, oral hypoglycemic agents; TG, total glycerides; UA, urine albumin; Hb, hemoglobin; Hct, hematocrit; UPCR, urine protein-to-creatinine ratio; eGFR, estimated glomerular filtration rate.

We analyzed the proportion of eGFR progression events during the follow-up period among patients with CKD stages 1–5 in different BMI groups. We also executed further subgroup analysis of the proportion of eGFR progression among patients with early-stage CKD (stages 1–3a) and late-stage CKD (stages 3b–5) in different BMI groups.

### Correlation between BMI and CKD progression among patients with CKD stages 1–5

**Figure 2:**
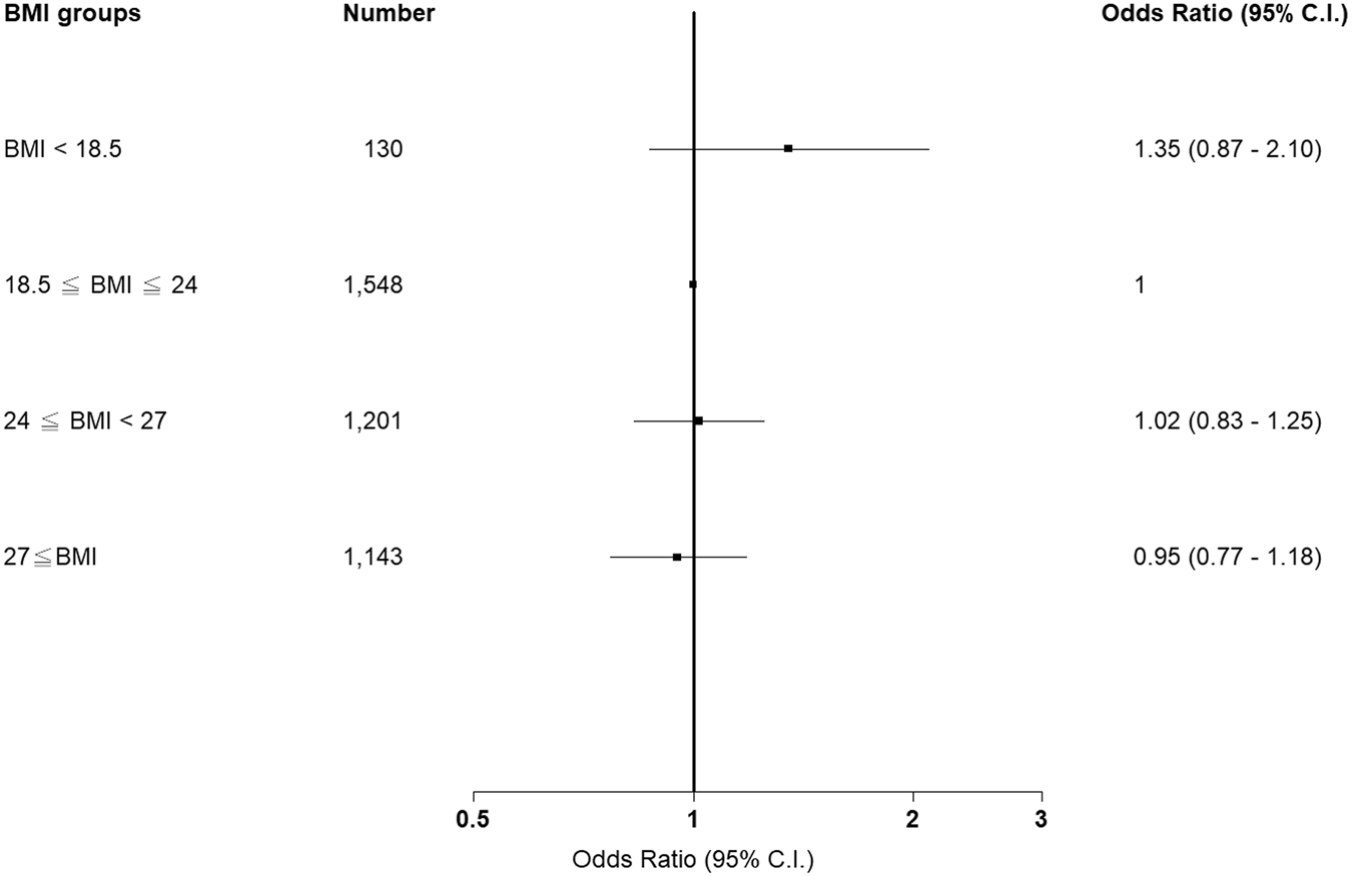
Association between body mass index and the risk of estimated glomerular filtration rate progression among patients with chronic kidney disease stages 1–5.

Table 4 presents the proportion of eGFR progression events in patients with CKD stages 1–5. The study outcomes are presented as ORs, and the normal group was used as the reference group to calculate the OR for each group. The underweight group exhibited the highest proportion of events (25%) compared with the normal (19%), overweight (19%), and obesity (18%) groups. The ORs of eGFR progression events were 1.44 (0.95, 2.18), 0.99 (0.81, 1.2), and 0.95 (0.78, 1.16) in the underweight, overweight, and obesity groups, respectively. After adjusting for age, sex, previous diabetes, CAD, stroke, cancer, high blood pressure, Charlson score, TB, COPD, ACEI, ARB, fibrate, smoking, alcohol consumption, betel nut chewing, baseline UPCR, and baseline eGFR, we observed that the OR was 1.35 (0.87, 2.10) in the underweight group compared with 1.02 (0.83, 1.25) and 0.95 (0.77, 1.18) in the overweight and obesity groups, respectively (Fig. 2).

**Table 4:** Association between BMI and the risk of eGFR progression among patients with CKD stages 1–5.

BMI group	No. of events	Proportion (%)	Study outcome, OR (95% CI)			
			Unadjusted	*P* value	Adjusted	*P* value
BMI < 18.5 (*n* = 130)	33	25	1.44 (0.95, 2.18)	0.0851	1.35 (0.87–2.10)	0.1855
18.5 ≤ BMI < 24 (*n* = 1548)	296	19	1	—	1	—
24 ≤ BMI < 27 (*n* = 1201)	227	19	0.99 (0.81, 1.20)	0.8838	1.02 (0.83–1.25)	0.8428
27 ≤ BMI (*n* = 1143)	210	18	0.95 (0.78, 1.16)	0.6232	0.95 (0.77–1.18)	0.6517

Models were adjusted for age, sex, DM, CAD, Stroke, cancer, hypertension, Charlson score, TB, COPD, ACEI, ARB, fibrate, smoking, alcohol consumption, betel nut chewing, UPCR and eGFR.

OR, odds ratio; CI, confidence interval; BMI, body mass index; DM, diabetes mellitus; CAD, coronary artery disease; CKD; chronic kidney disease; UPCR, urine protein-to-creatinine ratio; eGFR, estimated glomerular filtration rate.

### Correlation between BMI and CKD progression among patients with early-stage CKD (stages 1–3a)

**Figure 3:**
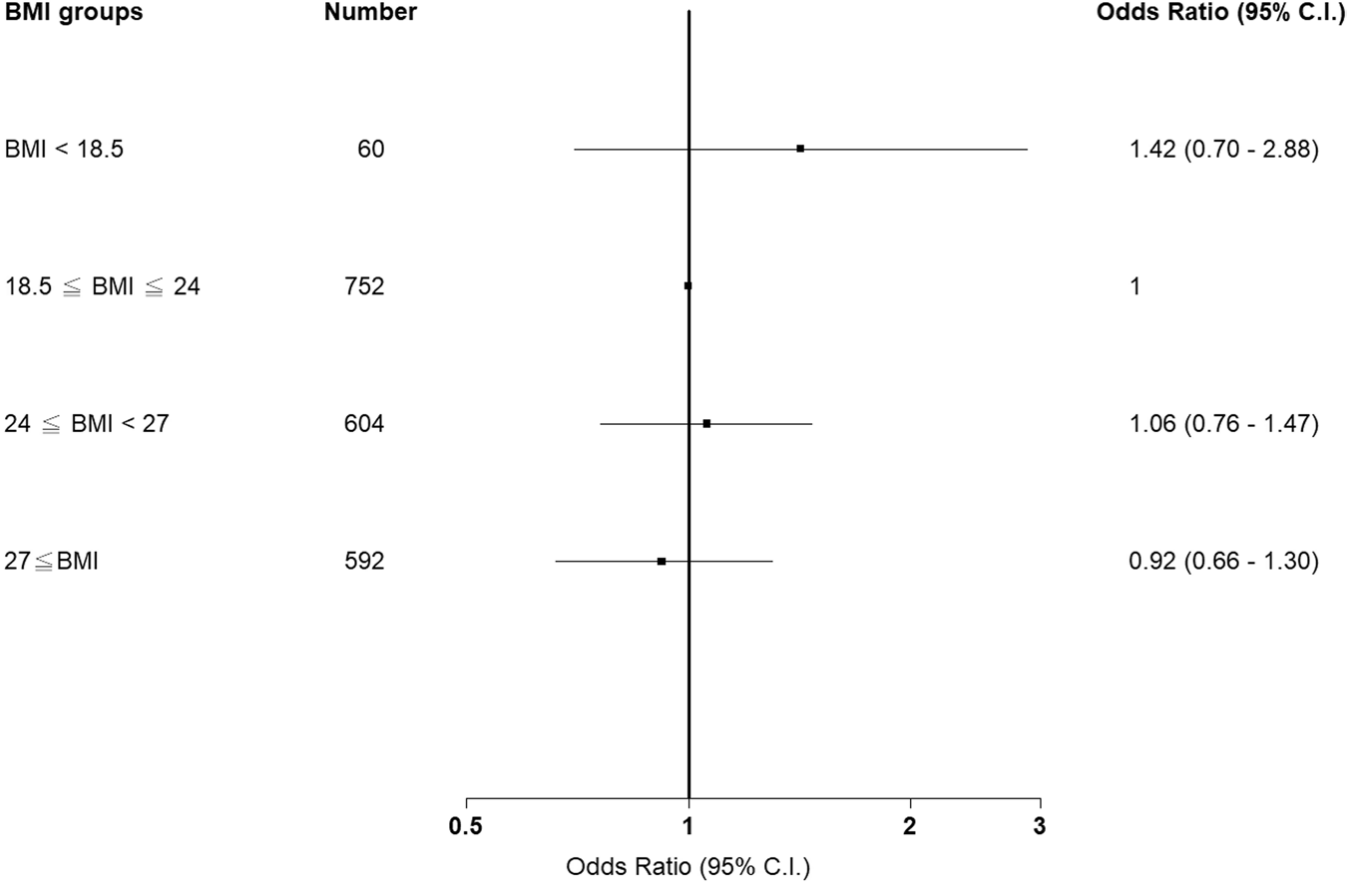
Association between body mass index and the risk of estimated glomerular filtration rate progression among patients with chronic kidney disease stages 1–3a.

Table 5 presents the proportion of eGFR deterioration events in patients with CKD stages 1–3a. The study outcomes are presented as ORs, and the normal group was used as the reference group to calculate the OR for each group. The underweight group exhibited the highest proportion of eGFR deterioration events (20%) compared with the normal (13%), overweight (13%), and obesity (12%) groups. The ORs of eGFR progression events were 1.67 (0.86, 3.25), 0.98 (0.71, 1.34), and 0.91 (0.66, 1.26) in the underweight, overweight, and obesity groups, respectively. After adjusting for age, sex, previous diabetes, CAD, stroke, cancer, high blood pressure, Charlson score, TB, COPD, ACEI, ARB, fibrate, smoking, alcohol consumption, betel nut chewing, baseline UPCR, and baseline eGFR, we determined that the ORs were 1.42 (0.70, 2.88), 1.06 (0.76, 1.47), and 0.92 (0.66, 1.30) in the underweight, overweight, and obesity groups, respectively (Fig. 3).

**Table 5:** Association between BMI and the risk of eGFR progression among patients with CKD stages 1–3a.

BMI group	No. of events	Proportion (%)	Study outcome, OR (95% CI)			
			Unadjusted	*P* value	Adjusted	*P* value
BMI < 18.5 (*n* = 60)	12	20	1.67 (0.86, 3.25)	0.1327	1.42 (0.70–2.88)	0.3325
18.5 ≤ BMI < 24 (*n* = 752)	98	13	1	—	1	—
24 ≤ BMI < 27 (*n* = 604)	77	13	0.98 (0.71, 1.34)	0.877	1.06 (0.76–1.47)	0.7474
27 ≤ BMI (*n* = 592)	71	12	0.91 (0.66, 1.26)	0.5687	0.92 (0.66–1.30)	0.6435

Models were adjusted for age, sex, DM, CAD, Stroke, cancer, hypertension, Charlson score, TB, COPD, ACEI, ARB, fibrate, smoking, alcohol consumption, betel nut chewing, UPCR and eGFR.

OR, odds ratio; CI, confidence interval; BMI, body mass index; DM, diabetes mellitus; CAD, coronary artery disease; CKD; chronic kidney disease; UPCR, urine protein-to-creatinine ratio; eGFR, estimated glomerular filtration rate.

### Correlation between BMI and CKD progression among patients with late-stage CKD (stage 3b–5)

**Figure 4:**
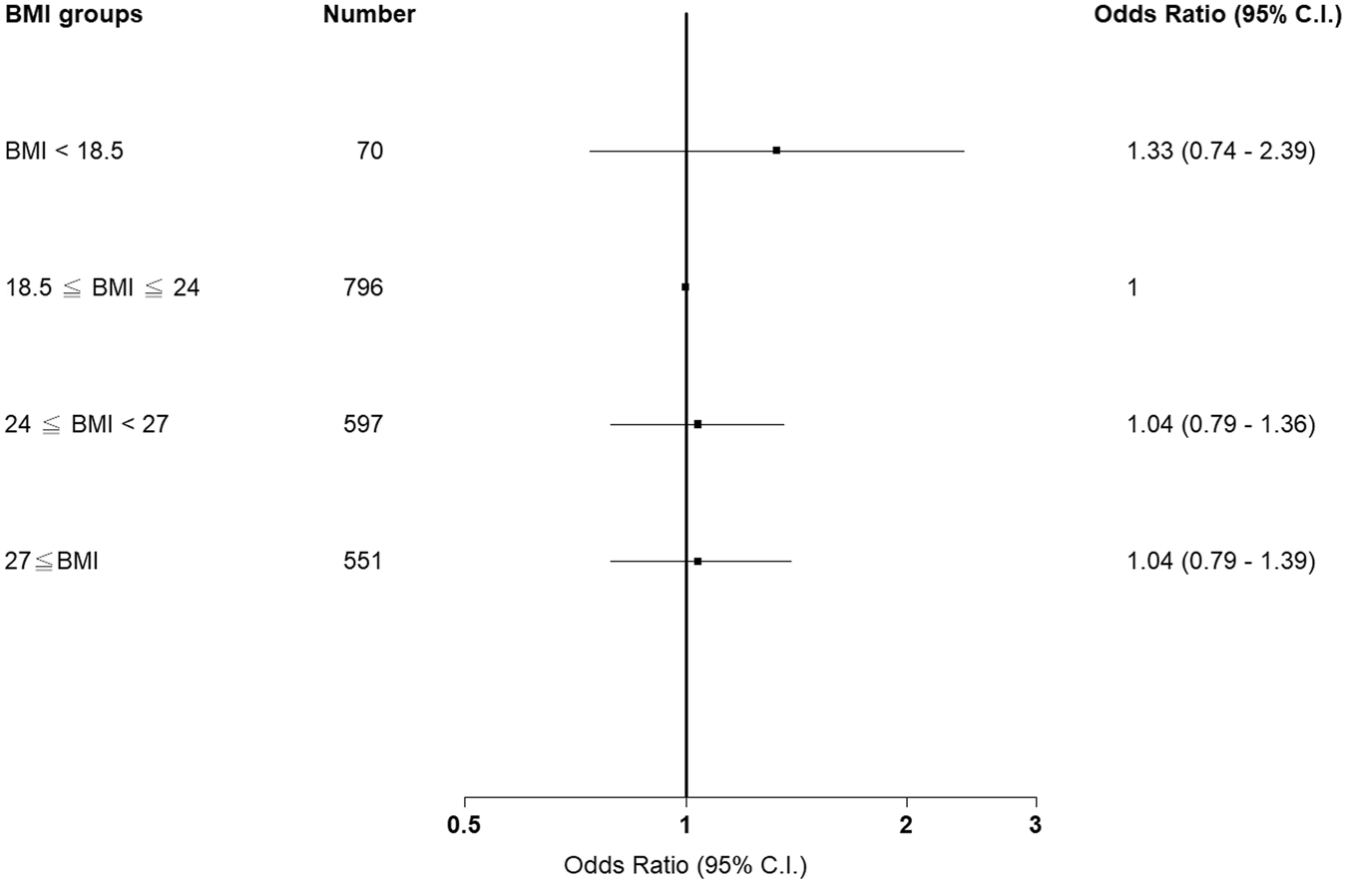
Association between body mass index and the risk of estimated glomerular filtration rate progression among patients with chronic kidney disease stages 3b–5.

Table 6 presents the proportion of eGFR deterioration events in patients with CKD stages 3b–5. The study outcomes are presented as ORs, and the normal group was used as the reference group to calculate the OR for each group. The underweight group exhibited the highest proportion of eGFR deterioration events (30%) compared with the normal (25%), overweight (25%), and obesity (25%) groups. The ORs of the eGFR progression events were 1.29 (0.76, 2.21), 1.01 (0.79, 1.30), and 1.02 (0.79, 1.31) in the underweight, overweight, and obesity groups, respectively. After adjusting for age, sex, previous diabetes, CAD, stroke, cancer, high blood pressure, Charlson score, TB, COPD, ACEI, ARB, fibrate, smoking, alcohol consumption, betel nut chewing, baseline UPCR, and baseline eGFR, we observed that the ORs were 1.33 (0.74, 2.39), 1.04 (0.79, 1.36), and 1.04 (0.79, 1.39) in the underweight, overweight, and obesity groups, respectively (Fig. 4).

**Table 6:** Association between BMI and the risk of eGFR progression among patients with CKD stages 3b–5.

BMI group	No. of events	Proportion (%)	Study outcome, OR (95% CI)			
			Unadjusted	*P* value	Adjusted	*P* value
BMI < 18.5 (*n* = 70)	21	30	1.29 (0.76, 2.21)	0.3452	1.33 (0.74–2.39)	0.3415
18.5 ≤ BMI <24 (*n* = 796)	198	25	1	—	1	—
24 ≤ BMI <27 (*n* = 597)	150	25	1.01 (0.79, 1.30)	0.9146	1.04 (0.79–1.36)	0.7866
27 ≤ BMI (*n* = 551)	139	25	1.02 (0.79, 1.31)	0.8832	1.04 (0.79–1.39)	0.7641

Models were adjusted for age, sex, DM, CAD, Stroke, cancer, hypertension, Charlson score, TB, COPD, ACEI, ARB, fibrate, smoking, alcohol consumption, betel nut chewing, UPCR and eGFR.

OR, odds ratio; CI, confidence interval; BMI, body mass index; DM, diabetes mellitus; CAD, coronary artery disease; CKD; chronic kidney disease; UPCR, urine protein-to-creatinine ratio; eGFR, estimated glomerular filtration rate.

## Discussion

In this prospective cohort study, we evaluated the characteristics of a CKD cohort according to various BMI categories. Subsequently, we investigated the association between BMI and the risk of eGFR decline among patients with different CKD stages. The highest prevalence of overweight and obesity was observed among men and the working age group (45–64 years old) in both early- and late-stage CKD. Previous studies conducted on the Japanese^24,25^ and Malay populations^26^ have demonstrated a male sex-specific association between BMI and kidney disease; similarly, from our baseline data, we observed a higher male prevalence among overweight and obese CKD patients. The mechanism underlying the male sex-specific association between higher BMI and CKD remains unclear; however, several studies have identified that BMI reflects visceral fat more efficiently in men than in women^27,28^. Generally, men exhibit a higher risk of kidney disease and develop the disease earlier in life than women because of hormonal and lifestyle influences^29–32^.

We observed a significantly higher prevalence of DM and hypertension among overweight and obese patients with CKD (both early- and late-stage CKD). This observational association might not represent cause and effect; since obesity itself is associated with various adverse sequelae from metabolic syndrome, as well as from comorbidities including DM and hypertension^33,34^, and all these conditions are associated with CKD. Patients with early-stage CKD with a lower BMI exhibited significantly higher cancer prevalence; however, non-significantly higher prevalence was observed in patients with late-stage CKD. This might also not represent a causal relation; nevertheless, many studies have revealed a bidirectional association between CKD and cancer^35,36^. Cancer patients with lower BMI exhibited associated nutritional disturbances and tended to have reduced renal function status from nutritional and specified therapies. A Korean study reported a significantly higher risk of CKD and proteinuria among cancer survivors^37^.

**Figure 5:**
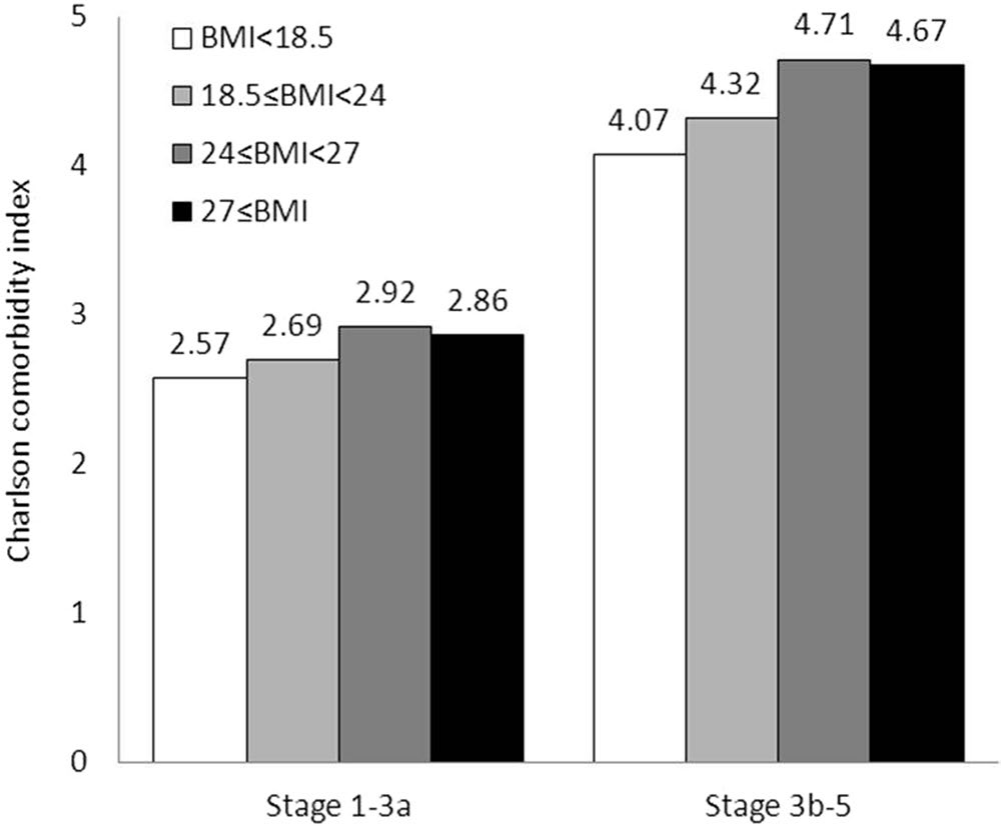
Charlson comorbidity score according to body mass index in patients with chronic kidney disease stages 1–5.

Furthermore, both CKD and ESRD are higher risks from a number of malignancies^38,39^. We calculated the Charlson scores at different CKD stages, which did not differ significantly among patients with early-stage CKD in different BMI categories. However, the median Charlson score increased significantly among overweight and obese patients with late-stage CKD (Fig. 5), which demonstrates the presence of more comorbidity among these patients.

The initial CKD stages did not differ significantly with BMI among patients with early-stage CKD (Table 2). However, in patients with late-stage CKD, the prevalence of the initial stages of CKD differed significantly according to BMI (Table 6), with a higher prevalence of CKD stages 3b and 4 observed in overweight and obese patients. We observed a significantly higher number of patients with CKD stage 5 to be underweight (BMI < 18.5). The reason for this finding is unknown, and additional studies are required to confirm whether any nutritional and concurrent comorbidity might play a role in such lower BMI prevalence among these patients. An obesity paradox was supposed for stage 5 CKD, because low BMI represents more uremia-associated inflammatory cachexia and high BMI represents fewer uremic consequences and more favorable health^40–42^. More emphasis on improved and adequate nutrition is required for these patients with advanced-stage CKD compared with control obesity in healthy patients.

A follow-up analysis revealed non-significantly increased CKD progression events among underweight patients compared with overweight and obese patients in both early and late CKD stages. This observed association persisted after adjustment for age, sex, previous diabetes, CAD, stroke, cancer, high blood pressure, Charlson score, TB, COPD, ACEI, ARB, fibrate, smoking, alcohol consumption, betel nut chewing, baseline UPCR, and baseline eGFR, and it was consistently present in the subgroup analysis among all patients with CKD. Our results are consistent with the so-called obesity paradox^43^ among patients with CKD; we found non-significantly higher kidney disease progression events among underweight patients compared with overweight and obese patients in all stages of CKD. Although several mechanisms have been proposed for patients with late-stage CKD^42^, we observed the same paradox among patients with early-stage CKD, which might be explained by the older age and higher cancer prevalence among patients with lower BMI. In patients with late-stage CKD, lower BMI was associated with poor nutritional status^42^ or higher prevalence of metabolically obese normal-weight individuals with a higher comorbidity burden^44^.

Our study results are consistent with those of other population studies. Data from a nationally representative cohort of US veterans with eGFR < 60 mL/min indicated a U-shaped association between BMI and the risk of renal progression^20^, with deteriorating outcomes observed in individuals with BMI < 25 kg/m^2^ and BMI ≥ 35 kg/m^2^; these data demonstrate that overweight or mild obesity (30–35 kg/m^2^) results in the most favorable outcomes and that in advanced CKD stages (eGFR < 30 mL/min), even morbid obesity (BMI ≥ 35 kg/m^2^) is not associated with adverse outcomes. A similar U-shaped association between an increased risk of progressive CKD and lowest BMI levels was noted in a large population-based cohort study in Israel^45^.

A retrospective study in the Taiwanese general population reported that waist-to-height ratio (WHtR), rather than BMI, increased as the prevalence of CKD increased^46^. Other studies have reported that WHtR and waist circumference, but not BMI, were associated with mortality in patients with CKD and ESRD^47,48^. Although the recent global definition uses BMI as a standard measure of obesity, obesity is affected by muscle mass, peripheral and abdominal adipose tissue mass, and bone; thus, the results should be considered with the condition^49^. Central obesity has been proved to be more vulnerable to metabolic syndrome and obesity-related diseases, whereas peripheral obesity and higher muscle mass appear protective^50–52^. BMI failed to represent central obesity because of the variation in individual body composition and contribution. This explains the nonsignificant association between BMI and CKD progression through all stages of CKD in our study.

Our study has several limitations. Because we studied a prevalent cohort of patients with CKD, we could not determine the effects of obesity on incident CKD. We used the study participants’ personal identities to link health care databases, and because the NHI database is based on the reporting data system and does not include the population not under medical health care, the study result may not represent the whole population; however, the missing population is negligible. We used only BMI to determine obesity, which may not be an ideal marker of obesity among our cohort; nevertheless, because BMI is generally accepted as a predominant index to establish obesity in clinical practice, our results have direct clinical relevance. Because the blood and urine samples of study participants were collected from individual hospitals and sent to the research center, the use of different equipment and personnel of individual hospitals may have resulted in measurement errors. Furthermore, we did not determine the influence of low and high BMI on mortality outcomes.

## Materials and Methods

### Ethics statement

The study was reviewed and approved by the institutional ethical committee of Taipei Medical University - Shuang Ho Hospital (TMU-JIRB 201204036), Tri-Service General Hospital (TSGHIRB100-05-197), Cardinal Tien Hospital (TMU-JIRB 201204035), Changhua Christian Hospital (CCHIRB 20405), Kaohsiung Medical University Chung-Ho Memorial Hospital (KMUHIRB 20120019), Kaohsiung Chang Gung Memorial Hospital (101-1096B), National Cheng Kung University Hospital (A-ER-101-117) and China Medical University Hospital (DMR101-IRB2-273(CR-1)). After a complete explanation of the study, written informed consent was obtained from all participants. All clinical and biological samples were collected after patient consent. All the study methods were in accordance with the guidelines approved by the joint institutional review board and aforementioned governmental regulations.

### Study population

We conducted a multicenter, longitudinal cohort study using data from the Epidemiology and Risk Factors Surveillance of CKD database (2008–2013) managed by the Bureau of Health Promotion, Ministry of Health and Welfare, Taiwan. After excluding patients with incomplete or missing data, we linked the biochemical laboratory data to the NHIRD from 2001 to 2013. The same medical laboratory criteria and protocol have been used in our study hospitals, and the serum creatinine levels derived from different hospitals can be compared and standardized with each other. In this study, we measured CKD progression at the individual level. In addition, the patients were reexamined in the same hospital to control the individual variation. All patients provided informed consent before data collection.

### Measurements and variable definitions

The patients’ demographic, clinical, and health-related behavior data were collected using a structured questionnaire. The questionnaire collected data on age, sex, cigarette smoking, alcohol consumption, betel nut chewing, personal and family comorbid conditions, and medication use. Physical examination included anthropometry, blood pressure measurement, pulse rate measurement, and systemic examination. Height was measured in centimeters by using a wall-mounted measuring tape, and weight was measured in kilograms by using a digital scale (SECA, model 782 2321009; Vogel & Halke, Germany). BMI was classified into the following groups: <18.5 kg/m^2^ (underweight), 18.5–23.9 kg/m^2^ (normal), 24–26.9 kg/m^2^ (overweight), and ≥27 kg/m^2^ (obesity). Glycemia, blood pressure, and lipid control conditions were classified as intensive and poor. Proteinuria status was determined using the urine protein-to-creatinine ratio (UPCR). CKD was defined according to the Kidney Disease Outcomes Quality Initiative guidelines^53^ and was evaluated using the estimated glomerular filtration rate (eGFR), which was calculated using the Chronic Kidney Disease-Epidemiology Collaboration equation: eGFR (mL/min/1.73 m^2^) = 141 × min (SCr/ƙ, 1)α × max (serum creatinine/ƙ, 1) − 1.209 × 0.993Age × 1.018 (if female) and × 1.159 (if black), where SCr denotes the serum creatinine level (mg/dL), ƙ = 0.7 (for women) and 0.9 (for men), α = −0.329 (for women) and −0.411(for men), min denotes the minimum of SCr/ƙ or 1, and max denotes the maximum of SCr/ƙ or 1^54^. CKD was classified as follows: CKD stage 1, eGFR ≥ 90 mL/min/1.73 m^2^ and the presence of kidney damage (i.e., proteinuria dipsticks ≥1+, UPCR ≥ 150, or urine albumin-to-creatinine ratio [UACR] ≥30); CKD stage 2, eGFR = 60–89 mL/min/1.73 m^2^ and the presence of kidney damage (i.e., proteinuria dipsticks ≥1+, UPCR ≥ 150, or UACR ≥ 30); CKD stage 3a, eGFR = 45–59 mL/min/1.73 m^2^; CKD stage 3b, eGFR = 30–44 mL/min/1.73 m^2^; CKD stage 4, eGFR = 15–29 mL/min/1.73 m^2^; and CKD stage 5, eGFR < 15 mL/min/1.73 m^2^^ 55^. Renal progression was defined as an average eGFR decline by more than 5 mL/min/1.73 m^2^ per year or into the dialysis stage^56^.

### Statistical analysis

Consistent with the study hypothesis, all analyses were stratified according to BMI. We examined BMI as quartiles: <18.5, 18.5–23.9, 24–26.9, and ≥27 kg/m^2^. The characteristics of different BMI groups were compared using the chi-squared test for categorical variables and ANOVA for continuous variables. The odds ratio (OR) (95% confidence interval) of CKD was calculated for each BMI category. Next, we explored the data for confounding and effect modification in stratified analyses. After adjusting for all covariates, we used the multivariate logistic model with stepwise variable selection models to evaluate the association between BMI and eGFR decline. In our subsequent multivariate modeling, we considered covariates including age; sex; comorbid conditions such as diabetes mellitus (DM), stroke, and cancer; the Charlson comorbidity index; use of antihypertensive medications (e.g., ACEI/ARB and loop diuretics) within the previous 1 year; and baseline CKD stage. The SAS statistical package (Version 9.3, SAS Institute Inc., Cary, NC, USA) was used for all statistical tests. Results with P < 0.05 were considered statistically significant.

## Conclusions

In conclusion, the definition and classification of obesity among patients with CKD should be intensively re-determined, because misdiagnosis can lead to inappropriate clinical decisions and might deteriorate patients’ prognosis. The anthropomorphic measures alternate to BMI should be established from randomized controlled clinical trials among the CKD population.
