# Author Correction: Relationship between body mass index and renal function deterioration among the Taiwanese chronic kidney disease population

**DOI:** 10.1038/s41598-020-59783-w

**Published:** 2020-02-13

**Authors:** Tian-Jong Chang, Cai-Mei Zheng, Mei-Yi Wu, Tzu-Ting Chen, Yun-Chun Wu, Yi-Lien Wu, Hsin-Ting Lin, Jing-Quan Zheng, Nain-Feng Chu, Yu-Me Lin, Sui-Lung Su, Kuo-Cheng Lu, Jin-Shuen Chen, Fung-Chang Sung, Chien-Te Lee, Yu Yang, Shang-Jyh Hwang, Ming-Cheng Wang, Yung-Ho Hsu, Hung-Yi Chiou, Senyeong Kao, Yuh-Feng Lin

**Affiliations:** 1 Graduate Institute of Life Sciences, National Defense Medical Center, Taipei, Taiwan; 2 Performance Appraisal Section, Secretary Office, Shuang Ho Hospital, Taipei Medical University, Taipei, Taiwan; 3 Division of Nephrology, Department of Internal Medicine, School of Medicine, College of Medicine, Taipei Medical University, Taipei, Taiwan; 4 Division of Nephrology, Department of Internal Medicine, Shuang Ho Hospital, Taipei Medical University, Taipei, Taiwan; 5 Graduate Institute of Clinical Medicine, College of Medicine, Taipei Medical University, Taipei, Taiwan; 6 Institute of Epidemiology and Preventive Medicine, College of Public Health, National Taiwan University, Taipei, Taiwan; 7 School of Nursing, College of Nursing, Taipei Medical University, Taipei, Taiwan; 8 Kidney Disease Prevention Foundation, Taipei, Taiwan; 9 Department of Ophthalmology, Tri-Service General Hospital, National Defense Medical Center, Taipei, Taiwan; 10 Department of Critical Care Medicine, Shuang Ho Hospital, Taipei Medical University, Taipei, Taiwan; 11 School of Public Health, National Defense Medical Center, Taipei, Taiwan; 12 Department of Medical Education and Research, Kaohsiung Veterans General Hospital, Kaohsiung, Taiwan; 13 School of Public Health, College of Public Health and Nutrition, Taipei Medical University, Taipei, Taiwan; 14 Division of Nephrology, Department of Medicine, Fu-Jen Catholic University Hospital, School of Medicine, Fu-Jen Catholic University, Taipei, Taiwan; 15 Division of Nephrology, Department of Medicine, Tri-Service General Hospital, National Defense Medical Center, Taipei, Taiwan; 16 School of Public Health, Graduate Institute of Clinical Medical Science, China Medical University, Taichung, Taiwan; 17 Division of Nephrology, Kaohsiung Chang Gung Memorial Hospital, Chang Gung Medical University, Kaohsiung, Taiwan; 18 The Division of Nephrology, Changhua Christian Hospital, Changhua, Taiwan; 19 Division of Nephrology, Department of Medicine, Kaohsiung Medical University Hospital, Kaohsiung, Taiwan; 20 Division of Nephrology, Department of Internal Medicine, Cheng Kung University Medical Center, Tainan, Taiwan

Correction to: *Scientific Reports* 10.1038/s41598-018-24757-6, published online 02 May 2018

The original version of this Article contained an error in Affiliation 3, which was incorrectly given as ‘Department of Internal Medicine, School of Medicine, College of Medicine, Taipei Medical University, Taipei, Taiwan’. The correct affiliation is listed below:

Division of Nephrology, Department of Internal Medicine, School of Medicine, College of Medicine, Taipei Medical University, Taipei, Taiwan

This error has now been corrected in the HTML and PDF versions of the Article.

